# Intramural Hematoma of the Esophagus Complicating Severe Preeclampsia

**DOI:** 10.1155/2017/6304194

**Published:** 2017-05-18

**Authors:** Simone Garzon, Giovanni Zanconato, Nicoletta Zatti, Giuseppe Chiarioni, Massimo Franchi

**Affiliations:** ^1^Department of Surgical, Odontostomatological and Maternal and Child Sciences, University of Verona, Piazzale L.A. Scuro 10, 37134 Verona, Italy; ^2^Department of Medicine and Gastroenterology, University of Verona, Piazzale L.A. Scuro 10, 37134 Verona, Italy

## Abstract

Intramural hematoma of the esophagus is a rare injury causing esophageal mucosal dissection. Forceful vomiting and coagulopathy are common underlying causes in the elderly population taking antiplatelets or anticoagulation agents. Acute retrosternal pain followed by hematemesis and dysphagia differentiates the hematoma from other cardiac or thoracic emergencies, including acute myocardial infarction or aortic dissection. Direct inspection by endoscopy is useful, but chest computed tomography best assesses the degree of obliteration of the lumen and excludes other differential diagnoses. Intramural hematoma of the esophagus is generally benign and most patients recover fully with conservative treatment. Bleeding can be managed medically unless in hemodynamically unstable patients, for whom surgical or angiographic treatment may be attempted; only rarely esophageal obstruction requires endoscopic decompression. We report an unusual case of esophageal hematoma, presenting in a young preeclamptic woman after surgical delivery of a preterm twin pregnancy, with a favorable outcome following medical management.

## 1. Introduction

Intramural hematoma of the esophagus (IHE) is a rare condition in which bleeding starts within the submucosal layer causing the hematoma formation and the dissection of the esophageal wall [1, 2].

IHE is considered part of a spectrum of esophageal injuries that include local mucosal tears (Mallory-Weiss syndrome), full-thickness rupture (Boerhaave's syndrome), and dissecting intramural hematoma [3]. Although these syndromes are usually associated with severe vomiting, dissecting IHE is not always associated with an increase in intraesophageal pressure. Other underlying causes of submucosal bleeding can be any one of the following: coagulopathy and abnormal hemostasis, trauma, and portal hypertension. IHE has severe implications but an excellent prognosis when managed conservatively [4, 5].

We report the case of a dissecting intramural hematoma of the esophagus acutely complicating a preeclamptic woman shortly after Cesarean section.

## 2. Case Presentation

A 37-year-old G2P1 woman of Indian origin was admitted in the Obstetrical Department of the University Hospital of Verona with a 32-week dichorionic twin gestation. She complained of moderate dyspnea (SpO2 98%) and sudden ankle swelling; her blood pressure was high and had significant proteinuria (>30 mg/mmol on spot protein-creatinine). Obstetric history included a term vaginal delivery and a laparoscopic salpingectomy for ectopic pregnancy. She had conceived the index pregnancy after an in vitro fertilization and embryo transfer (IVF-ET) in India. In her country of origin, she had gone through regular antenatal visits, receiving vaginal progesterone, anticoagulant therapy with LMWH for multiple venous thromboembolism risk factors, and levothyroxine due to a pregestational autoimmune hypothyroidism. Prophylactic cerclage had also been placed in India at 14 weeks where she was started on a daily low-dose aspirin regimen.

At admission, treatment was started with oral labetalol 100 mg every 8 hours and a 2-day betamethasone course for fetal lung maturation. Aspirin was discontinued, while LMWH was kept, due to multiple risk factors: age (37 years), twin pregnancy, IVF/ART, and preeclampsia. Twenty-four-hour proteinuria was 2,1 g/d and preeclampsia was confirmed. Platelet count showed a reduction from 125 × 10^9^/L at admission to 98 × 10^9^/L. Ultrasound evaluation showed a normal growth, normal amniotic fluid, and Doppler indices of uterine blood flow for both fetuses.

At 33 weeks of gestation, the patient was delivered with a C-section under general anesthesia due to an uncontrolled rise of the blood pressure failing to respond to treatment (160/100 mmHg) and a further platelet count reduction (91,000/dL). Bleeding amounted to 600 mL. After C-section, no evidence of coagulopathy was observed with stable platelet count. Hemoglobin levels (11.5 g/dL) as well as other coagulation indexes remained in the normal range. The newborns weighted 2090 g and 1830 g and were transferred to the Neonatology Intensive Care unit to be treated for prematurity.

**Figure 1:**
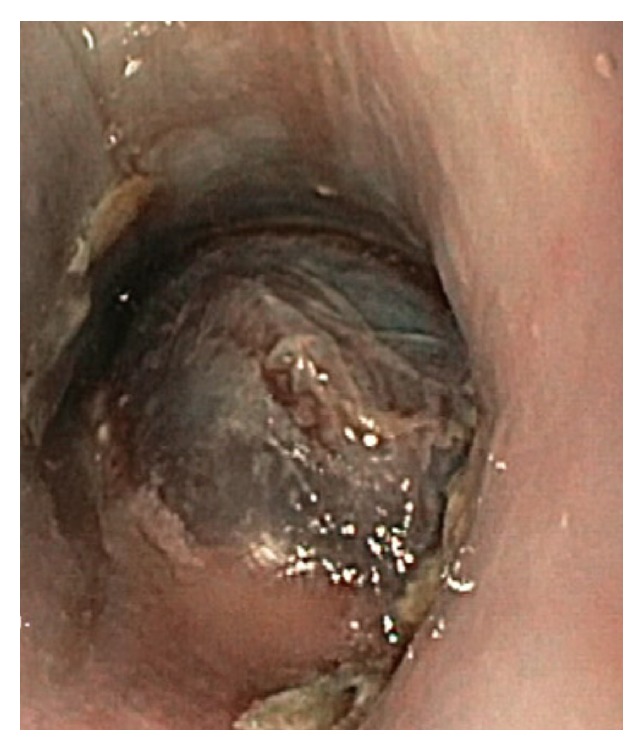
Endoscopic view of a bluish mass obliterating the lumen of the proximal esophagus.

After Cesarean, a sudden increase of blood pressure (175/110 mmHg) was observed and intravenous labetalol was started along with magnesium sulphate for eclampsia prevention. LMWH was continued to prevent postpartum and postoperative DVT. Treatment effectively lowered the blood pressure and obtained a stable condition, with the patient only complaining of moderate heartburn and occasional vomiting. Accordingly, H2 receptor antagonists therapy was started. On the first postoperative day, the patient suddenly complained of a right side retrosternal pain extending to the shoulder blade, nausea, vomiting, and dysphagia. Occurrence of HELLP syndrome was ruled out since platelet count and liver enzymes were normal and no signs of hemolysis were recorded. ECG and chest X-ray were both negative. The following day hematemesis was observed which warranted the indication of a gastroscopy. In the initial phase of the endoscopic evaluation, an eccentric mass was seen, completely obliterating the lumen of the esophagus, which prompted the end of the procedure (Figure 1).

**Figure 2:**
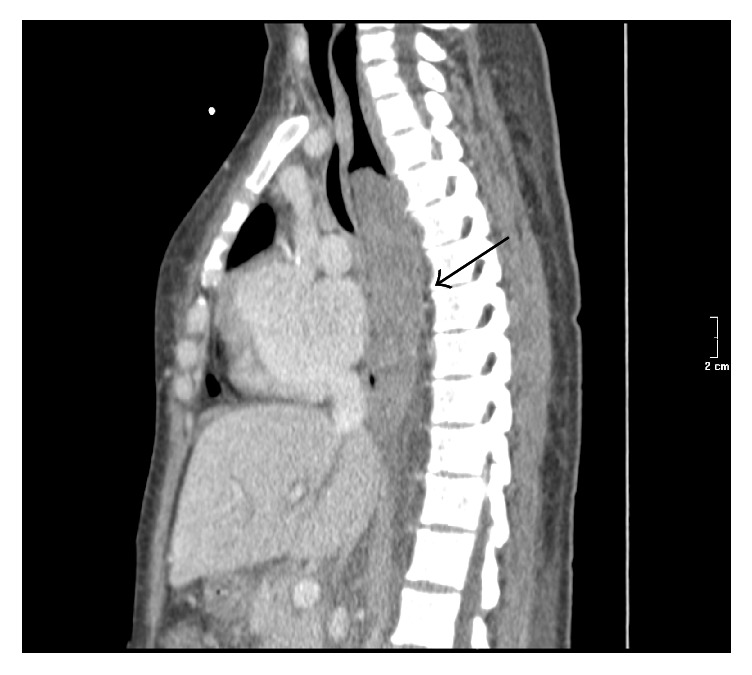
Second post-C-section day chest CT sagittal view of the esophageal hematoma (black arrow) infiltrating the posterior wall.

A chest CT scan showed bilateral pleural effusion and confirmed an intramural hematoma of the posterior esophageal wall, extending 15 cm caudally (Figure 2); no active bleeding was seen.

The patient was transferred to ICU where she remained hemodynamically stable, with no sign of perforation and no mediastinal involvement. A conservative management was chosen: supportive care and parenteral nutrition, administration of proton pump inhibitors to decrease gastric acid production, and broad-spectrum antibiotic therapy.

The chest CT control two days later showed no modification of the hematoma with no active bleeding and improvement of the pleural effusion.

Nine days after the initial diagnosis and eleven days after C-section, a new episode of hematemesis required a repeat CT scan. This time a reduction of the hematoma was seen with concomitant blood collection in the stomach. Gastroscopy was indicated, blood content aspirated, and the active bleeding controlled with local therapy. The patient was transfused with 2 units of packed red cells.

After this episode, the patient was discharged from ICU and transferred to a surgical ward where conservative treatment was continued until restarting of food intake; the subsequent course was uncomplicated.

**Figure 3:**
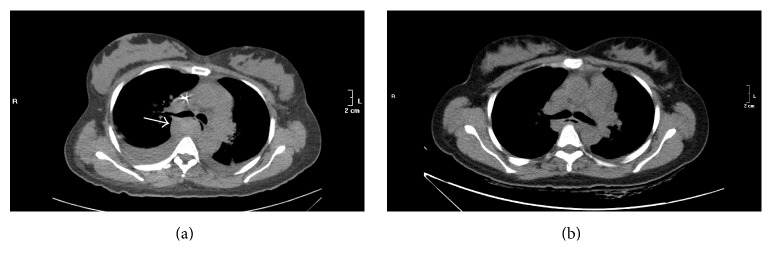
Chest CT transverse view of (a) hematoma (white arrow) displacing the esophageal lumen on 2nd day after C-section. (b) Normal esophageal anatomy and lumen patency after full recovery, 17 days after C-section.

A control chest CT, 17 days after C-section, showed an open lumen, a reduction of the thickness of the esophageal wall, and an almost complete resolution of the parietal hematoma (Figure 3).

The patient was discharged asymptomatic 20 days after the initial diagnosis under antihypertensive and antacid therapy. A detailed chart including medications, laboratories results, images, and procedures throughout hospitalization is presented in Table 1.

## 3. Discussion

Pathogenesis of intramural esophageal bleeding leading to hematoma formation and submucosal dissection is often unclear. Several causes have been proposed: emetogenic, traumatic, related to aortic disease, and coagulopathic; in few instances, the cause remains undetermined. Severe bleeding results in proximal or distal intramural dissection, as the hematoma develops concentrically or eccentrically, as in the present case [5]. About one-fifth of patients appear to have a spontaneous origin, although this may be associated with an underlying predisposition: abnormal pressure changes within the esophagus or a coagulation disorder [6].

Although the direct cause was not clear, we do not consider emesis a relevant contributing factor in the present case. The patient complained of little vomiting, not strong enough, in our opinion, to raise the intrathoracic pressure and to produce intramural trauma and hemorrhage. Instead, we believe the following were precipitating factors: coagulopathy secondary to LMWH treatment and platelet reduction in association with high blood pressure during the episode of severe preeclampsia. Even though no case of IHE in association with gestational hypertension has ever been described, it is a well-known fact that preeclampsia carries an increased risk of bleeding and of several hemorrhagic complications such as liver, renal, and intracranial hematoma [7–10].

We also tend to exclude the iatrogenic trauma from intubation during general anesthesia since no abnormal manoeuver was reported by the anesthesiologist.

Clinical presentation of this case with chest pain and dysphagia matches the observations of other authors: gradually increasing chest pain (66–84%) localized in the retrosternal or epigastric region and odynophagia/dysphagia (26–59%), exacerbated by swallowing [5]. Differently from the Mallory-Weiss syndrome where the debut symptom is usually hematemesis, this complaint is an infrequent initial manifestation of IHE. According to other studies, the clinical triad of retrosternal pain, difficulty in swallowing, and hematemesis is present in just one-third of cases [11]. Hematemesis occurs when the intramural hematoma expands and the mucosa ruptures. Blood loss is usually moderate, and only 10% of the patients will require a transfusion [12].

In any preeclamptic woman presenting with chest pain, other complications which need to be ruled out are HELLP syndrome, congestive cardiac failure, liver hematoma, or liver rupture. The association of chest pain with dysphagia and hematemesis helps the clinician in reaching the correct diagnosis of this rare situation which has a 2 : 1 female preponderance and a mortality rate of 7–9% after either surgical or medical treatment [13].

Endoscopy is considered the first-line diagnostic tool and it revealed the esophageal lesion in our case. However, this technique has some disadvantages when compared with chest CT scan since it is an invasive procedure which may not reveal the abnormality in the absence of a mucosal tear and may further damage the esophageal wall. Chest CT has the advantage of being noninvasive and finds indication to confirm the diagnosis and to exclude active bleeding; besides the esophageal wall, it explores the aorta and other mediastinal structures, thus excluding other thoracic processes [5].

After the diagnostic confirmation, the hematoma was treated conservatively and resolved in less than 3 weeks, a length of time necessary to obtain the spontaneous drainage, the complete healing of the mucosal tear, and the recovery of a normal esophageal peristalsis [5, 14]. Although most bleeding in IHE can be managed medically, surgical drainage and repair of the laceration or therapeutic angiography may become an urgent indication in those cases of massive hemorrhage and hemodynamically unstable patients [15].

In conclusion, although a rare event, IHE may unexpectedly complicate the pregnant state. Diagnosis is not always simple, since chest pain is a presenting symptom found in other conditions, particularly in a preeclamptic woman. Medical treatment is associated with full recovery in most cases and surgical or endoscopic interventions are rarely required.

## Figures and Tables

**Table 1:** Time chart of the entire hospitalization period.

	Admission			C-section	Pain symptoms	IHE diagnosis		Bleeding episode		Discharge
*Treatment*										
Aspirin	Stop									
HLMW	•	•	•	•	•	•				
Mg				•	•					
Betamethasone		•	•							
*Laboratory*										
Hb (gr/dL)	11.7	12.1	12.2	11.2	9.5	8.7	7.8	6.5	8.4	11.2
INR		0.84	0.83	0.84	0.79	0.87	0.94	1	1.06	1.01
PLT (10^3^/dL)	125	114	122	91	103	131	127	264	307	353
AST/ALT (UI/L)	—/39	46/35	—/36	—/39	—/24	—/18		—/16		38/43
BP	140/95	130/90	140/90	160/100	175/110	120/80	<140/90	<140/90	<140/90	<140/90
24 h U proteins		2.1 gr								0.89 gr
*Imaging/procedures*										
Chest CT						•	•	•	•	
Endoscopy						•		•		
Blood transfusion								•		
*Day*	*1*	*2*	*3*	*8*	*9*	*10*	*12*	*19*	*25*	*30*
